# Implementing a "publish, then review" model of publishing

**DOI:** 10.7554/eLife.64910

**Published:** 2020-12-01

**Authors:** Michael B Eisen, Anna Akhmanova, Timothy E Behrens, Diane M Harper, Detlef Weigel, Mone Zaidi

**Affiliations:** 1 eLifeCambridgeUnited Kingdom

**Keywords:** scientific publishing, peer review, preprints, research communication, research assessment

## Abstract

From July 2021 eLife will only review manuscripts already published as preprints, and will focus its editorial process on producing public reviews to be posted alongside the preprints.

The growing popularity of preprints has enabled researchers to make their papers freely and immediately available to anyone with an internet connection. Many eLife authors were early adopters of preprinting, and support within our community continues to expand: a recent internal analysis showed that nearly 70% of papers under review at eLife were already available on bioRxiv, medRxiv or arXiv.

This is a major milestone. It means that for all practical purposes eLife is no longer a publisher: rather, eLife is now an organization that reviews and certifies papers that have already been published. We welcome this moment, and the long-awaited opportunity it provides to replace the traditional "review, then publish" model developed in the age of the printing press with a "publish, then review" model optimized for the age of the internet. Henceforth, eLife will focus its editorial and technology development efforts on bringing this new model to life in a way that benefits authors, readers, potential readers, the broader research community and the public.

Here we detail changes to eLife policy and practice that are the first major steps in this process. First, we are shifting to exclusively reviewing manuscripts that have been posted as preprints. Second, we are refocusing our editorial processes away from deciding what papers should be published, and towards transforming preprints into "refereed preprints" that include a public assessment of the work prepared by our reviewers and editors.

While we are moving quickly to seize this opportunity, not all changes will be immediate. We want to make sure we get our processes and policies right, we want to make sure authors who work with us in developing and refining a new system are not penalized for doing so and, most of all, we want to bring our entire community along with us into this new publishing world.

We are, in particular, ever mindful that the authors who entrust us to review their papers still have to operate in a world where journal citations are the currency of careers. Thus, while our long-term goal is to move science away from the use of journal titles as the primary measure of the quality of research, until an alternative takes hold, we will still be selecting papers to be "published" in eLife.

With the embrace of bioRxiv and medRxiv by eLife authors, we feel our community is ready for us to become the first major journal to move to only reviewing preprints, and to work with us to reoptimize peer review for this new era of science publishing.

## Exclusively reviewing preprints

Our first step towards our goal of exclusively reviewing preprints is to make the posting of preprints the default. If any paper we are planning to send out to peer review is not already on a preprint server, we will post it to bioRxiv or medRxiv (as appropriate) on behalf of the authors. We expect that many of our authors who previously did not post preprints did so out of inertia rather than opposition, and will now do so.

However, we are aware that some potential authors have real concerns about posting preprints, and others are restricted from doing so for various reasons. We would like to take some time to understand and, where possible, mitigate these concerns, especially in areas where adoption of preprints has lagged behind. Therefore, for the next six months, we will give authors of papers under review the option to opt out of posting a preprint, and will instead ask them to explain their reasons.

Once we feel we understand and have addressed concerns about preprinting that are in our power to address, we will move to only review papers that are already available as preprints. It will still be possible for authors to request a waiver of this policy in exceptional circumstances, but we expect these to be rare.

## Preparing and posting public reviews

One of the biggest challenges we face in implementing this new system is that peer reviews are typically not written for a public audience. We learned from Preprint Review (https://twitter.com/PreprintReview), our current opt-in system for reviewing preprints, that informing reviewers that their reviews would be posted on bioRxiv only rarely led them to write their reviews in a different way. We also learned that it is very difficult to inspire new editorial and reviewer practices when they are only employed for a small percentage of articles. We have therefore concluded that we have to go all in for this system to succeed. Hence, starting immediately, we are instructing our editors and reviewers to prepare public reviews for all manuscripts under consideration.

We have created a new review template, updated our instructions for reviewers to capture what we think should and should not be included in public reviews, and have modified our editorial processes to focus on their production. In the coming months, we will be working closely with our editors, reviewers, authors and readers to improve both the process by which public reviews are created, and their utility to the diverse audiences we hope to reach.

## Posting public reviews

While we will prepare public-facing reviews for every paper under review at eLife, authors will retain a degree of control over when these are posted. For papers that receive a favorable decision from the journal, and where the authors elect to proceed with eLife, the public review will be posted to the appropriate preprint server within three weeks (to allow authors time to prepare a response).

However, if our editors decide a paper is not appropriate for eLife, we will, for now, allow authors to postpone the posting of the public review until the paper is published elsewhere. Allowing such delays will address a potential major obstacle to adoption – author fears that negative public reviews will prejudice their ability to publish their work in a journal – while ensuring that authors cannot permanently avoid dealing with issues that may have arisen during our review.

Proceeding in this manner will allow us to work constructively with authors while we develop and refine our review processes, and introduce the system to the wider scientific community. However, as we get better at producing constructive public reviews, and as authors get more comfortable with both the idea and our implementation of it, we expect to move towards the rapid posting of all reviews, irrespective of the associated publishing decision.

## Publication as curation

As mentioned above, our long-term goal is to move away from the use of journal titles as the primary measure of the quality of research in science and medicine. However, we know that journal titles remain important for many researchers as they pursue their careers. Thus, while we are developing alternatives, for the foreseeable future, we will continue to select a subset of the papers we review for "publication" in eLife.

The process will be similar to what it is today (see our author guide for details). After the reviews on the paper have been submitted, the editors and reviewers will consult with each other to decide: (i) what they wish to convey in the public review; (ii) if the paper is appropriate for publication in eLife. The authors will then be sent a copy of the public review as well as a letter outlining the reasons for the decision regarding publication in eLife: if a revision is invited, this decision letter will list the issues that need to be addressed before the manuscript can be accepted.

Accepted manuscripts will be handled exactly as they are today: they will be formatted, assigned a DOI and posted on the eLife website, with, if appropriate, associated magazine content. Thus, from the outside, eLife will still be publishing papers. However, the decision to accept a manuscript for publication in eLife will now become an initial act of curation on a paper that has already been published by the authors. In other words, "selected for inclusion in eLife" will become a sort of badge that we attach to papers.

While pragmatism dictates that we maintain this legacy of print era publishing, our long term goal is to render it irrelevant as well as obsolete. We have already begun working on ways to downweight journal title as the main indicator of manuscript quality. We plan, for example, to introduce richer evaluation metrics that can be published alongside articles – both those that we publish in the journal, and those that we review as preprints – ending the absurd process of bouncing from journal to journal until a paper is accepted. We are also developing a new platform for interacting with preprints and public reviews (https://sciety.org/).

## The future

All of these changes, if widely adopted, will create a version of the current publishing system that is more efficient, effective and transparent. But the real opportunities – and challenges – will come from the more radical and dramatic changes to science publishing that will be possible once we finally break free of the "one paper, one journal, one publication model" that still dominates the field.

There is no reason for papers to be reviewed only once, or by only one entity. The review process should involve multiple voices and go on for as long as the work is relevant. It should be possible for reviews to be written by anyone with something useful to say about a work – not just the people who have been selected by a journal or other entity. And one can imagine all manner of more useful ways to organize and curate the literature than just putting papers into a single "journal".

But we also have to recognize the potential perils of such a system. We do not want the evaluation of science to become any more of a popularity contest than it already is, and we want to make sure that the process is as fair and free of bias as humanly possible. We cannot count on the wisdom of the crowd to solve these problems for us. So as we move towards this new system, we also reiterate our commitment to observe how it is impacting everyone involved in science and medicine, and to always be willing to fight for changes that make research communication better for all.

